# Potential biomechanical risk factors on developing lead knee osteoarthritis in the golf swing

**DOI:** 10.1038/s41598-022-27160-4

**Published:** 2022-12-31

**Authors:** Sung Eun Kim, Nicole Segovia Pham, Jae Hyeon Park, Amy Ladd, Jangyun Lee

**Affiliations:** 1 Department of Orthopaedic Surgery, Stanford University, Stanford, CA USA; 2 Motion & Gait Analysis Laboratory, Lucile Packard Children’s Hospital, Palo Alto, CA USA; 3 Department of Rehabilitation Medicine, Hanyang University Guri Hospital, Guri-Si, Gyeonggi-Do Korea; 4 Department of Orthopedic Surgery, 6-01 Dongguk University Ilsan Hospital, 27 Dongguk Ro, Ilsandong-Gu, Goyang-Si, 10326 Gyeonggi-Do Korea; 5 Department of Orthopaedic Surgery, Seoul National University College of Medicine, Seoul, Korea

**Keywords:** Risk factors, Biomedical engineering

## Abstract

The load on the lead knee joint during a golf swing is greater than that observed during gait. However, current evidence regarding golf swing biomechanics for risks associated with knee osteoarthritis (OA) is limited. Therefore, this study investigated golf swing styles associated with knee adduction and abduction moments, which are considered to be crucial loading regions of the medial and lateral compartments of knee OA, respectively. Thirteen professional male golfers performed five shots using a 5-iron club, and their swings were recorded using a motion capture system with two force platforms for the feet. A regression analysis was performed to calculate the correlation coefficients between the peak knee adduction and abduction moments of the lead leg and varus/valgus angle, toe-out angle, stance width, weight transfer, and shoulder sway. Swinging with a narrower stance width at address (*r* =  − 0.62, *p* = 0.02) with more weight shift (*r* = 0.66, *p* = 0.014) and shoulder sway (*r* = 0.79, *p* = 0.001) towards the target during the downswing were associated with a higher peak knee adduction of the lead leg, whereas a greater valgus angle at address (*r* = 0.60, *p* = 0.03) was associated with a higher peak knee abduction of the lead leg. Based on these findings, we anticipate future research to support postural changes, particularly a wider stance width and restricted shoulder sway for golfers who are classified to be at high risk of developing medial compartment knee OA, as well as a lower valgus (tibial medial tilt) angle at address for those classified to be at high risk of developing lateral compartment knee OA.

## Introduction

Multidisciplinary biomechanical investigations to reduce the risk of knee osteoarthritis (OA) have been conducted in various academic fields, including rehabilitation medicine^1,2^, physiotherapy^3^, orthopedic surgery^4^, health sciences^5^, mechanical engineering^6^, and bioengineering^7^. These studies, primarily conducted for walking, consisted of identifying biomechanical risk factors^5^ as well as evaluating the effects of modifications on reduction of knee-joint loads^3^. Some golf swing modifications have been evaluated given that knee-joint loading is greater than gait and stair ascent^8–10^. Additionally, although efforts to develop preventive training that include personalized modifications^11^ and vision technology^7^ are currently available as treatment for gait, such advanced techniques are limited for golf. The increased popularity of golf, exceeding 60 million people worldwide during the COVID-19 pandemic^12^ suggests developing a preventative model that is applicable to not only gait but also golf swing. Such training tools potentially maximize the lifespans of the natural joints for persons who are classified to be at high risk for developing knee OA.

Current studies on the biomechanical risk factors of knee OA in golf identify the variables only at address (preswing)^9,13^, whereas golf swing consists of several phases from address, backswing, downswing, impact, follow-through to finish. This limited consideration presents a challenge for modeling prevention strategies because golf biomechanics (in-swing) may vary across golfers^14^. Moreover, primary knee loadings for the medial and lateral compartments of knee OA are knee adduction and abduction moments, respectively^9,15^; to the best of our knowledge, no risk factors have been identified for knee abduction moment in the golf swing. Diversely identified risk factors for both knee adduction and abduction, including in-swing variables, may therefore assist studies to develop preventative strategies in golf.

In previous studies, a lower toe-out angle of the lead foot (i.e., left foot for right-handed golfers and vice versa) and narrower stance width at golf address were suggested as biomechanical risk factors for medial compartment knee OA^9,13^. The present study hypothesizes that the varus angle and weight transfer during the golf swing may be additional biomechanical risk factors. In terms of the varus angle, a previous study found that walking with a greater varus angle (peak tibial lateral tilt relative to the laboratory during the stance phase) is correlated with a higher peak knee adduction moment^5^. For most cases, neutral alignment (0 ± 3°) is considered optimal for total-knee arthroplasty surgery to achieve neutral alignment and reduce knee pain^16^. Further, Ball and Best (2007a) found that there were two distinct styles of weight transfer, namely the front and reverse foot styles. The front foot style continues the center of pressure position towards the target through impact, whereas the reverse foot style moves the center of pressure position away from the target through impact^17^. Here, the former may lead to greater knee loading of the lead leg than the latter because the weight transfer reflects a greater ground reaction force applied through the lead foot. However, such potential relationships between the swing variables and knee loading have not been investigated in depth.

In this study, we investigated diverse and potential risk factors for developing knee OA based on the golf swing. We first examined whether the varus/valgus angle, toe-out angle, stance width, and greater weight transfer towards the target during the downswing were correlated with greater peak knee adduction and abduction moments of the lead leg. Additionally, we explored whether more pelvis and shoulder sway towards the target during the downswing were correlated with higher peak knee adduction and abduction moments so as to offer specific instructional suggestions should weight transfer be a risk factor. We hypothesized that greater peak knee adduction and abduction of the lead leg will be correlated with the varus/valgus angle, toe-out angle, stance width, and greater weight transfer as well as more pelvis and shoulder sway towards the target during the downswing.

## Methods

### Participants

Thirteen healthy professional male golfers (age: 29.0 ± 4.7 years; height: 177.4 ± 6.7 cm; mass: 76.1 ± 8.1 kg) participated in this study. The participants had no history of chronic pain or serious injuries within the last 6 months. This study was approved by the Institutional Review Board of Yonsei University, Korea, and all methods were performed in accordance with the relevant guidelines and regulations. All participants provided written informed consent.

### Procedures

**Figure 1:**
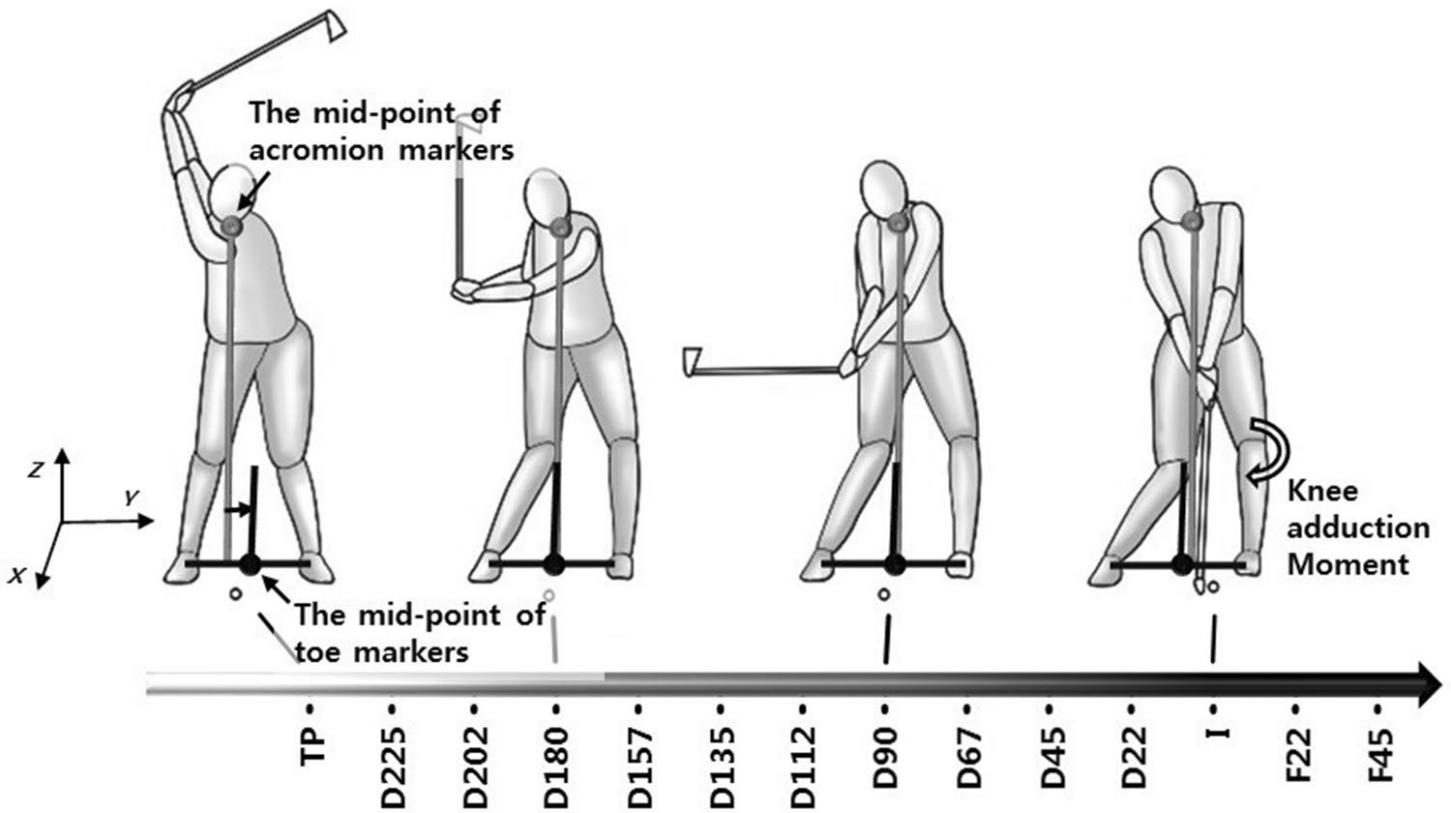
Fourteen sequential downswing events, peak knee adduction moment of the lead leg around impact, and shoulder position with respect to the mid-point of toe markers during the downswing. The horizontal axes show 14 sequential downswing events: transition of the pelvis (TP), downswing 225° (D225), downswing 202° (D202), downswing 180° (D180), downswing 157° (D157), downswing 135° (D135), downswing 112° (D112), downswing 90° (D90), downswing 67° (D67), downswing 45° (D45), downswing 22° (D22), impact (I), follow-through 22° (F22), and follow-through 45° (F45). *X*, *Y*, and *Z* front to back, side-to-side, and vertical axes, respectively. The direction of knee adduction moment of the lead leg (the tibia segment with respect to the thigh segment) and towards the target shoulder position (the mid-point of acromion markers with respect to the mid-point of toe markers) show positive direction.

The participants underwent 3D golf analysis with 35 reflective markers attached to the anatomical landmarks as per the Vicon Plug-in-Gait full-body model (Oxford Metrics, Oxford, UK)^18^. Additionally, four reflective adhesive tapes were attached to the 5-iron club (at the top of the clubhead, hosel, mid-point of the shaft, and immediately below the grip) and one reflective adhesive tape was placed on the golf ball to define the swing events (Fig. 1). The Vicon motion analysis system was used to capture golf kinematics using eight MX cameras recording at 250 Hz, which were integrated with two force platforms (AMTI, Watertown, MA, USA) embedded in the laboratory floor to collect ground reaction force data at 2000 Hz. The participants were instructed to perform their own typical warm-up before being asked to execute five straight golf shots using their 5-iron club off an artificial golf mat into a curtain placed 5 m away from the participant position. Among the various clubs, the 5-iron club was chosen because its swing mechanics is somewhere between those of the iron and driver shots, according to golf coaches, which may generally represent the swings for both the iron and driver shots. The coordinate system consisted of *X*-axis in the front-to-back direction, *Y*-axis in the side-to-side direction, and *Z*-axis in the vertical direction (Fig. 1).

### Data analysis

The captured raw data were smoothed with a Woltring filtering routine, with a 10 mm^2^ mean-squared error value^19^, which followed methods of a previous golf study that presented experiments with a 5-iron club^20^. The Vicon Nexus software was used to calculate knee adduction and abduction moments using an inverse dynamics approach. The knee adduction and abduction moments were normalized to the participants’ body mass^8,9,21^ and were calculated at the first peaks. There are generally two peaks for knee adduction during the golf swing, and the first peak was chosen in this study for consistent timing; an additional reason for this choice was that the timing of pain felt by golfers was reported to be around impact^21^, and the first peak occurs around impact while the second peak occurs near finish^8^. The first peak has also been consistently used in previous studies to examine modifications to reduce the peak knee adduction moment for golf swing^9,13^.

The lead leg’s tibial frontal plane lateral tilt (knee varus angle), where the tibia is defined as the line joining the centers of the knee and ankle joints relative to the laboratory vertical axis, was calculated at address and at first peak^5^. The lead foot external rotation (toe-out angle), where the foot was defined as the line joining the heel and 2nd metatarsal head markers relative to the laboratory anteroposterior axis, was calculated at address. The stance width at address was calculated in two ways: one used toe markers and the other used heel markers. In prior studies, the stance width was identified by the heel markers because golfers often have different degrees of toe-out angles^13^. However, in practice, the stance width is considered from the anterior view when coaches instruct golfers. Further, from the golfers’ perspective, the stance width at the toe is more visually noting than at the heel. Therefore, the stance width was added in this study using toe markers for practical application.

The center of pressure position (weight transfer), i.e., the weighted mean of the individual foot center of pressure, which is parallel with the laboratory mediolateral axis, was calculated^17,18,22^; it was expressed as a percentage of the distance between the trail foot (0%) and lead foot (100%)^17,22^. The pelvis position, where the pelvis is defined by the mid-point between the right and left anterior superior iliac spine markers, and shoulder position, where the shoulder is defined by the mid-point between the right and left acromion markers, parallel to the laboratory mediolateral axis (sway) were calculated with respect to the mid-points of the toe markers. The center of pressure as well as pelvis and shoulder positions were calculated at 14 sequential golf downswing events to employ statistical parametric mapping (SPM) during statistical analysis (see Fig. 1): transition of the pelvis^18,23^; shaft angles of 225°, 202°, 180°, 157°, 135°, 112°, 90°, 67°, 45°, and 22° with shaft parallel at the top being 270° in the frontal plane during the downswing (D225, D202, D180, D157, D135, D112, D90, D67, D45, and D22, respectively); impact (I); and shaft angles of 22° and 45° in the frontal plane during the follow-through (F22 and F45, respectively). SPM allows for testing correlation between a variable at a single event (peak knee adduction and abduction moments of the lead leg in this study) and a one-dimensional time-series variable (14 sequential downswing events of the center of pressure position and pelvis and shoulder positions in this study). Golfers use different swing tempos^24,25^; therefore, we used 14 sequential events instead of time trajectories. The transition of the pelvis was identified as the change in its rotational direction in the horizontal plane. Impact was defined at a distance closest between the golf ball and the mid-point of the clubhead along the mediolateral axis. The kinematics and kinetics of the five shots were averaged for each participant.

The directions of the lead tibial lateral tilt and lead foot external rotation angles relative to the laboratory were denoted as positive. The direction of the adduction moment of the lead tibial segment with respect to the lead thigh segment was denoted as positive (Fig. 1). Finally, the directions toward the target of the pelvis and shoulder positions with respect to the mid-points of the toe markers were also assigned positive values (Fig. 1).

### Statistical analysis

In order to investigate the correlations between the peak knee adduction and abduction moments of the lead leg and lead tibial lateral tilt, lead foot external rotation angle, and stance width, we performed either Pearson or Spearman correlation tests. Spearman correlation tests were performed if at least one of the Pearson correlation test assumptions such as normality (tested in this study using Shapiro–Wilk’s, skewness, and kurtosis assessments) and equal homoscedasticity were not satisfied. Furthermore, in terms of the center of pressure position and pelvis and shoulder positions, we performed SPM^26–29^ regression analysis using the open source (http://www.spm1d.org) MATLAB (Mathworks Inc., Natick, USA) code. The normality test was also conducted for SPM residuals. Non-parametric SPM regression analysis was performed if the assumption was not satisfied. For a particular event series where a significant correlation was found in SPM regression, either Pearson or Spearman (if the Pearson correlation test assumptions we mentioned above were not satisfied) correlation tests was performed in order to demonstrate the relationship between the independent variable and event-series of the dependent variable. The two-sided level of critical significance was set at *p* < 0.05.

## Results

**Figure 2:**
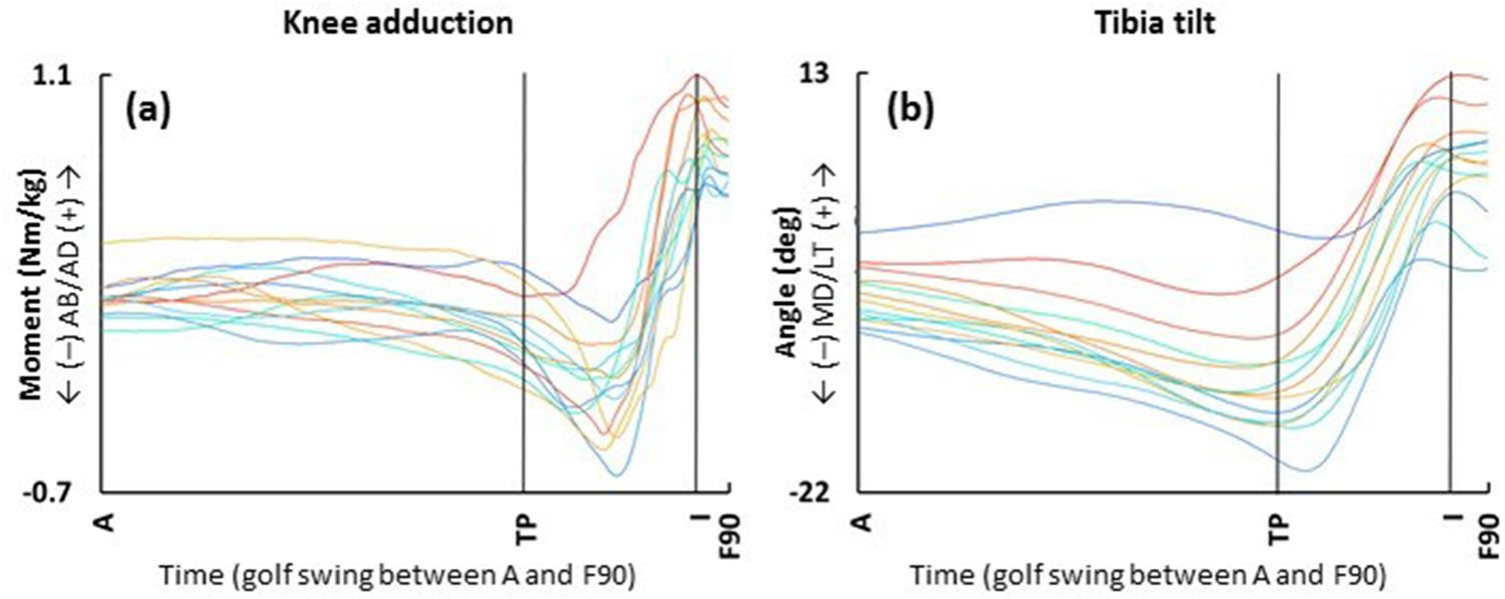
Frontal plane knee add/abduction moment and tibial medial/lateral tilt of the lead leg during the golf swing. Each line represents the mean trajectories of each participant over 5 shots for (**a**) knee add/abduction moment and (**b**) tibial medial/lateral tilt of the lead leg. The gradation of colors in warm and cool represent the high and low knee adduction moment at its peak, respectively. Direction of knee adduction moment (the tibia segment with respect to the thigh segment) and tibial lateral tilt (the tibia segment relative to laboratory) of the lead leg represents positive value. A: address, TP: transition of pelvis, I: impact, F90: follow-through 90°.

**Figure ****2** shows the lead knee add/abduction moment and lead tibia’s medial/lateral tilt during the golf swing.

### Knee adduction moment

**Figure 3:**
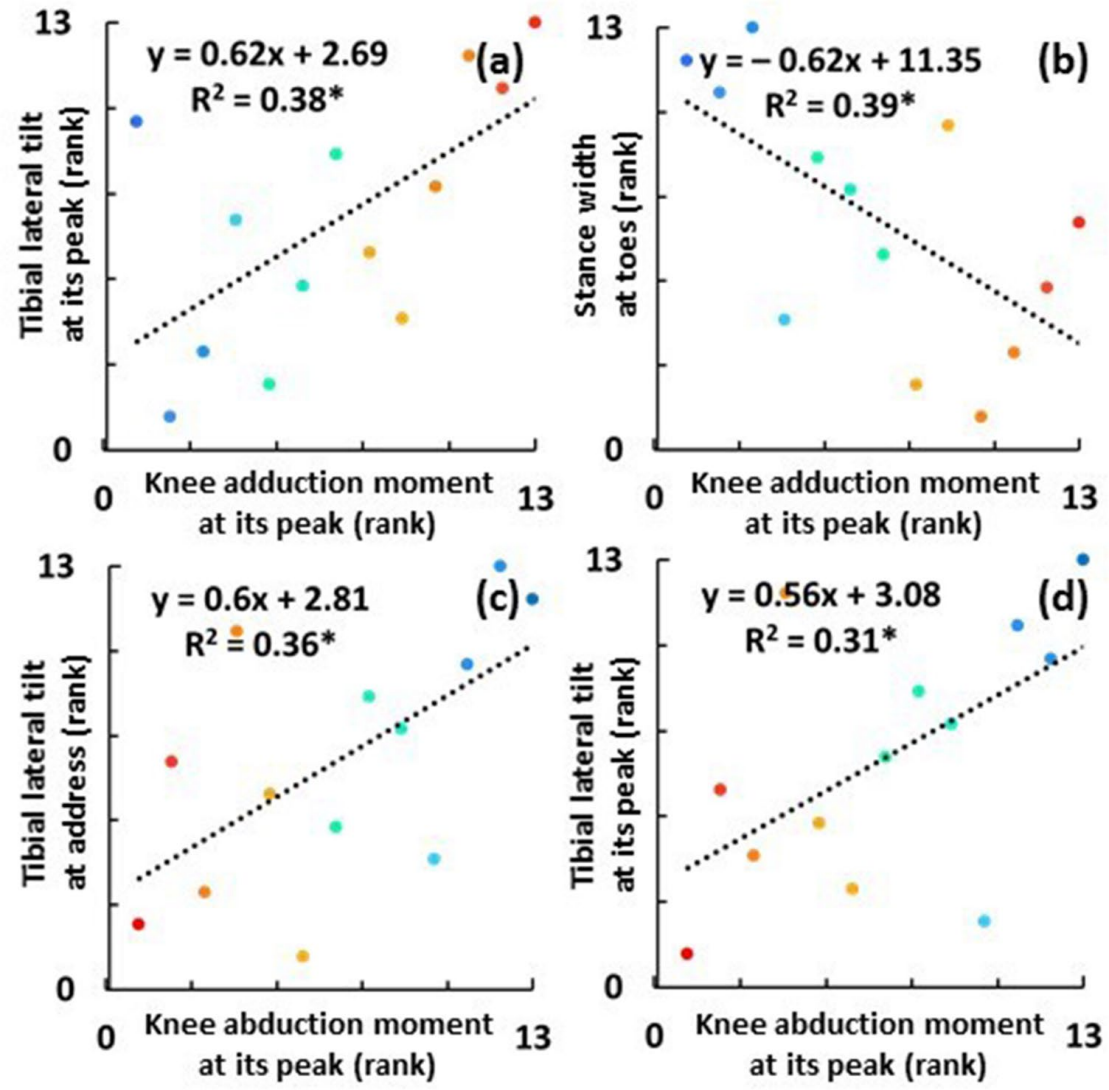
Spearman correlations between the peak knee adduction moment of the lead leg and (**a**) lead tibial lateral tilt at its peak and (**b**) stance width at address using toe markers and between the peak knee abduction moment of the lead leg and (**c**) lead tibial lateral tilt at address and (**d**) at its peak. Each dot represents the rank over mean value of each participant over 5 shots. The rank of the peak knee adduction moment is expressed in ascending order (1: low, 13: high), while that of the abduction is descending (1: high, 13: low) due to negative denotation. The gradation of colors in warm and cool represent the high and low knee add/abduction moments at its peaks, respectively. *significant at *p* < 0.05.

The Spearman correlation tests showed that several factors were associated with higher peak knee adduction moment of the lead leg (0.85 ± 0.15 Nm/kg which occurred at the club position of F14.96 ± 29.82°), including significantly greater lead tibial lateral tilt at its peak (5.91 ± 4.02° which occurred at the club position of D11.44 ± 87.16°) (*r* = 0.62, *p* = 0.03) (Fig. 3a) and narrower stance width at address, using toe markers (49.22 ± 3.6 cm) (*r* =  − 0.62, *p* = 0.02) (Fig. 3b). In contrast, the lead tibial medial tilt at address, lead foot external rotation at address, and stance width at address using heel markers were not significantly correlated (Table 1). The mean difference between the two stance widths (at toe and at heel) was 8.93 ± 3.27 cm.

**Table 1:** Spearman correlations between the peak knee adduction and abduction moments of the lead leg and lead tibial lateral tilt at address and its peak, lead foot external rotation at address (performed Pearson correlation), and stance width at address using toe and heel markers. In addition, Spearman correlations between the peak knee adduction moment of the lead leg and center of pressure position at F45 and shoulder position during the downswing (D225–D112).

Variables	Value	*r* value	*p* value	Club shaft (°)
**Lead knee adduction moment (+ : add)**				
Peak (Nm/kg)	0.85 ± 0.15			F14.96 ± 29.82
**Lead tibial lateral tilt relative to laboratory (+ : lateral)**				
Address (°)	− 5.56 ± 2.41	0.32	0.73	
Peak (°)	5.91 ± 4.02	0.62*	0.03	D11.44 ± 87.16
**Lead foot external rotation relative to laboratory (+ : external)**				
Address (°)	14.96 ± 5.84	0.03	0.998	
**Stance width using toe markers**				
Address (cm)	49.22 ± 3.6	− 0.62*	0.02	
**Stance width using heel markers**				
Address (cm)	40.29 ± 3.76	0.39	0.19	
**Center of pressure (+ : towards-target)**				
F45 (0–100%)	80.35 ± 10.31	0.66*	0.014	
**Shoulder position with respect to the mid-point of toe markers (+ : towards-target)**				
Averaged across D225–D112 (cm)	0.39 ± 2.0	0.79*	0.001	
**Lead knee abduction moment (+ : add)**				
Peak (Nm/kg)	− 0.28 ± 0.22			D251.9 ± 10.40
**Lead tibial lateral tilt relative to laboratory (+ : lateral)**				
Address (°)	− 5.56 ± 2.41	0.60*	0.03	
Peak (°)	5.91 ± 4.02	0.56*	0.046	D11.44 ± 87.16
**Lead foot external rotation relative to laboratory (+ : external)**				
Address (°)	14.96 ± 5.84	0.015	0.96	
**Stance width using toe markers**				
Address (cm)	49.22 ± 3.6	0.016	0.96	
**Stance width using heel markers**				
Address (cm)	40.29 ± 3.76	− 0.15	0.63	

F: follow-through, D: downswing. (mean ± SD).

*significant at *p* < 0.05.

**Figure 4:**
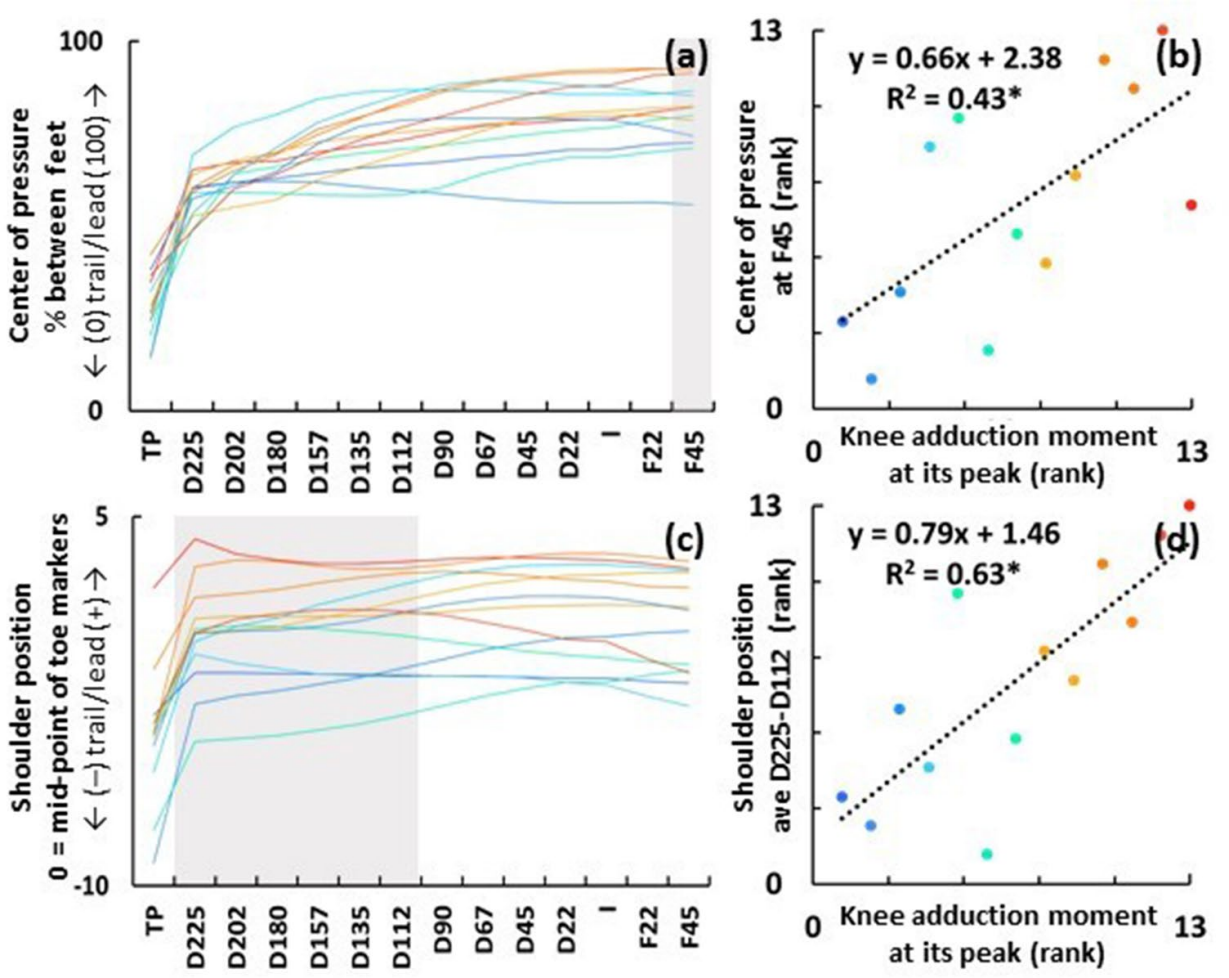
Center of pressure and shoulder positions during the downswing are displayed in the graphs (**a**) and (**c**), respectively. The gray areas represent the swing events that showed a significant correlation with the peak knee adduction moment of the lead leg in SPM regression analysis. Each line represents the mean trajectories of each participant over 5 shots. The gradation of colors in warm and cool represent the high and low knee adduction moment of the lead leg at its peak, respectively. The horizontal axes show 14 sequential downswing events (see Fig. 1). Graph (**b**) and (**d**) represent the Spearman correlations between the rank of peak knee adduction moment of the lead leg and center of pressure and shoulder positions (the rank of mean value averaged across the events showed a significant correlation in SPM regression), respectively. Direction of towards-target center of pressure position with respect to the trail foot (0%) has a positive value of maximum at lead foot (100%). Directions of towards-target shoulder position with respect to the mid-point of toe markers have a positive value. F45: follow-through 45°, D225: downswing 225°, D112: downswing 112°. *significant at *p* < 0.05.

SPM regression analysis revealed that the peak knee adduction moment of the lead leg was associated with the center of pressure position just after impact in the follow-through phase (F45) (*p* = 0.045) (Fig. 4a). The Spearman correlation showed that a greater towards-target center of pressure position at F45 (80.35 ± 10.31%) was associated with higher peak knee adduction moment of the lead leg (*r* = 0.66, *p* = 0.014) (Fig. 4b and Table 1).

SPM regression analysis revealed that the peak knee adduction moment of the lead leg was not associated with pelvis position during the downswing; however, it almost reached significance at *p* = 0.05 at transition of pelvis (TP).

Non-parametric SPM regression analysis revealed that the peak knee adduction moment of the lead leg was associated with shoulder position during the downswing (between D225 and D112) (*p* = 0.004) (Fig. 4c). The Spearman correlation showed that more towards-target shoulder position between D225 and D112 (0.39 ± 2.0 cm averaged across events then subjects) was associated with higher peak knee adduction moment of the lead leg (*r* = 0.79, *p* = 0.001) (Fig. 4d and Table 1).

### Knee abduction moment

The Spearman correlation tests showed that several factors were associated with higher peak knee abduction moment of the lead leg (− 0.28 ± 0.22 Nm/kg which occurred at the club position of D251.87 ± 10.40°), including significantly lesser lead tibial lateral tilt at address (− 5.56 ± 2.41°) (*r* = 0.60, *p* = 0.03) (Fig. 3c) and at its peak (5.91 ± 4.02°) (*r* = 0.56, *p* = 0.046) (Fig. 3d). In contrast, the lead foot external rotation at address (performed Pearson correlation) and stance width at address using both toe and heel markers were not significantly correlated (Table 1).

SPM regression analysis revealed that the peak knee abduction moment of the lead leg was not associated with the center of pressure position and pelvis and shoulder positions during the downswing (performed non-parametric for the pelvis) (Table 1).

## Discussion

This study investigated the relationships between the peak knee adduction and abduction moments of the lead leg and varus angle (lead tibial lateral tilt) at address and its peak, toe-out angle (lead foot external rotation) at address, stance width at address with respect to the toe and heel, weight transfer (weighted mean of individual center of pressure) during downswing, and towards-target pelvis and shoulder sway (pelvis and shoulder positions) during downswing to identify the potential biomechanical risk factors for developing knee OA based on the golf swing.

We found that the peak knee adduction moment of the lead leg occurring around impact was positively correlated with a greater varus angle around impact, narrower stance width with respect to the toe, greater weight transfer around impact, and more shoulder sway, whereas the toe-out angle and stance width with respect to the heel were not correlated. Further, higher peak knee abduction moment of the lead leg occurring just after transition of the pelvis was correlated with lesser varus angles at address and its peak, while all the other variables were not correlated.

It has been demonstrated by Levinger et al. that walking with a greater peak varus angle increases the load on the medial compartment of the knee joint—knee adduction moment^5^. Similarly, our findings suggest that motion of the tibial lateral tilt around impact (D11.44 ± 87.16°, Table 1) may be a potential biomechanical risk factor for developing medial compartment knee OA. In addition, radiographic evaluations have been presented in many gait analyses to investigate the anatomical factors that affect loading of the knee joint. These studies have found that the varus alignment was correlated with peak knee adduction moment during walking^30,31^. Therefore, in the future, the effects of individual anatomical factors on knee adduction moment during the golf swing may need to be investigated.

As hypothesized, the association between peak knee adduction moment of the lead leg and weight transfer immediately after impact was significant. This result suggests that weight transfer may be a potential biomechanical risk factor for developing medial compartment knee OA in the golf swing. Further, both the length of the moment arm (assessed by the magnitude of the varus angle) and magnitude of force (weight transfer) appear to be mechanisms contributing to knee adduction moment in the golf swing, whereas the moment arm has been found to be a primary contributor to knee adduction moment in gait^5^.

In a previous study, the pelvis and shoulder had large sways towards the target (approximately 15 and 7 cm, respectively) during the downswing^32^. If there is an association between the peak knee adduction moment of the lead leg and weight transfer during the downswing, it may be important to determine whether pelvis and shoulder sways towards the target during the downswing are also associated with peak knee adduction moment of the lead leg. As hypothesized, the shoulder sway towards the target (approximately 6 cm in our study) was positively correlated, while pelvis sway was not significantly correlated with peak knee adduction moment of the lead leg. Our result suggests that weight transfer may be reduced by restricting shoulder sway towards the target during the early downswing.

In terms of the variables at address, we found that a narrower stance width with respect to the toe at address was correlated with a higher peak knee adduction moment of the lead leg, whereas the toe-out angle and stance width at the heel, which were previously identified as risk factors^9,13^, were not correlated with the peak knee adduction moment of the lead leg. This was probably due to the difference in the technique of measurement of stance positions. The stance width at the toe generally represents the combination of toe-out angle and stance width at the heel. In practice, our results suggest that the stance width at the toe, regardless of the toe-out angle, may be more applicable than the previously identified risk factors as less adjustment is required for foot positioning as well as to enable the coaches’ anterior views of the golfers.

Finally, the lead leg’s lesser tibial lateral tilt at address and at its peak were associated with higher peak knee abduction moment. In practice, the tibial medial tilt at address is recommended to golfers for enhancing stability from a shoulder rotation of approximately 100° in both directions; however, our study suggests that it should not be recommended for golfers who are classified as being at high risk for lateral compartment knee OA. Furthermore, the peak lead tibial lateral tilt was correlated with both peak knee adduction and abduction moments. This result suggests that the peak lead tibial lateral tilt may be the primary feature (input) for modeling the framework to predict the peak knee adduction and abduction moments using vision-based technologies.

### Limitations of study

There are several limitations of this study. First, the regression analysis performed in this study only suggests a causal relationship between the variables. Therefore, further controlled experiments, such as evaluating the effects of modification on reduction of the peak knee adduction and abduction moments of the lead leg, are needed. Second, only professional golfers participated in this study, but further investigations are needed for recreational golfers since the swing techniques are different between recreational and professionals golfers^33^ and the swing kinematics vary greatly across recreational golfers and professional golfers^14^. Third, the development of medial compartment knee OA is mostly affected by varus alignment (as an anatomical factor) according to gait studies^30,31^. Therefore, investigations are needed that recruit golfers who have existing varus alignment. Although this study has the above limitations, conclusions may be extrapolated from a correlational study.

## Conclusion

We identified several potential golf swing biomechanical risk factors for both knee adduction and abduction moments. Based on our findings, for professional golfers who are classified to be at high risk for developing medial compartment knee OA, we potentially suggest a wider stance width at the toe and less shoulder sway towards the target during the early downswing. Further studies are required to evaluate the effects of a wider stance and restricted shoulder sway on reducing the peak varus angle, weight shift around impact, and peak knee adduction moment. Our findings indicate that golfers classified to be at high risk for developing lateral compartment knee OA would benefit from a less tibial medial tilt at address; however, further research is needed on the implications of increased peak varus angle and reduction of peak knee abduction moment. Ultimately, these several identified potential risk factors may inform prevention efforts of knee OA in golfers by suggesting modifications and their potential beneficial preventive effects.

## Data Availability

The datasets generated and/or analysed during the current study are not publicly available due to experiment protocol approved by the Institutional Review Board, but are available from the corresponding author on reasonable request.
